# Evaluation of machine learning methods with Fourier Transform features for classifying ovarian tumors based on ultrasound images

**DOI:** 10.1371/journal.pone.0219388

**Published:** 2019-07-26

**Authors:** José Martínez-Más, Andrés Bueno-Crespo, Shan Khazendar, Manuel Remezal-Solano, Juan-Pedro Martínez-Cendán, Sabah Jassim, Hongbo Du, Hisham Al Assam, Tom Bourne, Dirk Timmerman

**Affiliations:** 1 Department of Obstetrics and Gynecology, Virgen de la Arrixaca University Clinic Hospital, Murcia, Spain; 2 Health Sciences PhD program, Universidad Católica de Murcia UCAM, Murcia, Spain; 3 Department of Computer Science, Catholic University of Murcia (UCAM), Murcia, Spain; 4 Department of Administration and Economics, University of Sulaimani, Kurdistan, Iraq; 5 Department of Medicine; Obstetrics and Gynecology, Catholic University of Murcia (UCAM), Murcia, Spain; 6 Department of Obstetrics and Gynecology, Santa Lucía University General Hospital, Cartagena, Spain; 7 Department of Applied Computing, Buckingham University, Buckingham, United Kingdom; 8 Department of Cancer and Surgery, Queen Charlotte’s and Chelsea Hospital, Imperial College, London, United Kingdom; 9 Department of Development and Regeneration; Obstetrics and Gynecology, University Hospitals KU Leuven, Leuven, Belgium; Politechnika Krakowska im Tadeusza Kosciuszki, POLAND

## Abstract

**Introduction:**

Ovarian tumors are the most common diagnostic challenge for gynecologists and ultrasound examination has become the main technique for assessment of ovarian pathology and for preoperative distinction between malignant and benign ovarian tumors. However, ultrasonography is highly examiner-dependent and there may be an important variability between two different specialists when examining the same case. The objective of this work is the evaluation of different well-known Machine Learning (ML) systems to perform the automatic categorization of ovarian tumors from ultrasound images.

**Methods:**

We have used a real patient database whose input features have been extracted from 348 images, from the IOTA tumor images database, holding together with the class labels of the images. For each patient case and ultrasound image, its input features have been previously extracted using Fourier descriptors computed on the Region Of Interest (ROI). Then, four ML techniques are considered for performing the classification stage: K-Nearest Neighbors (KNN), Linear Discriminant (LD), Support Vector Machine (SVM) and Extreme Learning Machine (ELM).

**Results:**

According to our obtained results, the KNN classifier provides inaccurate predictions (less than 60% of accuracy) independently of the size of the local approximation, whereas the classifiers based on LD, SVM and ELM are robust in this biomedical classification (more than 85% of accuracy).

**Conclusions:**

ML methods can be efficiently used for developing the classification stage in computer-aided diagnosis systems of ovarian tumor from ultrasound images. These approaches are able to provide automatic classification with a high rate of accuracy. Future work should aim at enhancing the classifier design using ensemble techniques. Another ongoing work is to exploit different kind of features extracted from ultrasound images.

## Introduction

Ovarian tumors represent a very common diagnostic challenge for gynecologists. The majority prove to be benign (80-85%), and their maximum incidence is between 20 and 44 years of age [1]. Once an ovarian mass is detected, the clinician’s priority is to determine whether it is a benign or a malignant tumor, and to assess the options for optimal management. The risk of malignancy increases when the tumor is detected in prepuberal or postmenopausal women. The overall yearly incidence of malignant ovarian tumors is 9.9 per 100000 people, being the 6th cancer in women, the 5th cause of death by cancer in women, and the 1st cause of death by gynecological cancers in developed countries [2, 3]. Ultrasound examination has become the main technique for assessment of ovarian pathology and in the hands of an experienced examiner it has the highest performance for preoperative classification of malignant and benign ovarian tumors [4]. It should be noted that it is a non-invasive examination, there is no irradiation, and allows for assessing its size and suspicious signs such as presence of solid tissue, tumoral heterogeneity, presence and number of papillary structures, and presence of ascites. The reason for choosing the vaginal way to perform the ultrasound examination is to bring the ultrasound transducer closest to adnexal masses, such that it offers the highest image resolution and allows for a more sensitive Doppler signal.

There is no population screening because of its low incidence. Nevertheless, there are some special groups (such as women with a family history of ovarian cancer or other related cancers -breast, endometrial or colonic cancer) in which regular ultrasound examinations are recommended. The major limitation of ultrasound examination is that this is examiner-dependent and may have a large interobserver variability [5]. This is the reason why some researchers have developed tools to help professionals to interpret ultrasound images.

The most important pioneering study in this area resulted in the Risk of Malignancy Index (RMI), published in 1990 by Jacobs et al. [6], The RMI is based on the menopausal status of the patient, the serum Ca125 level, and a score based on the presence or absence of suspicious ultrasound features. They reported a sensitivity for cancer of 85% and a specificity of 97%.

Some years after this publication, the International Ovarian Tumor Analysis (IOTA) collaborative group published a consensus paper on Terms and Definitions to describe ultrasound features of benign and malignant ovarian tumors [7, 8], such as papillary projections, irregular internal walls in cystic lesions, presence of ascites or abnormal vascular flow. The IOTA group’s work [7, 8] reached an important international impact, because it was the first publication on standardized ovarian tumor examination. In 1999, this research group also published on Artificial Neural Networks (ANNs) [8] and compared the obtained results with subjective assessment, Logistic Regression (LR) models and the Risk of Malignancy Index, obtaining a sensitivity of 95.9% and specificity of 93.5% with ANNs. They continued with proposing and testing Simple Rules based on ultrasound features [9, 10], and determined that better results were obtained using the Simple Rules as a triage test and after that, a second stage test when Simple Rules yielded an inconclusive result, being the subjective assessment by the ultrasound examiner, which proved the best second stage test, obtaining a sensitivity of 91% and specificity of 93%. A recent meta-analysis [11] suggested that the preoperative characterization of any adnexal mass should incorporate the use of IOTA Simple Rules or the LR2 logistic regression model, especially for women of reproductive age, because it showed the best performance in validation studies with an overall sensitivity and specificity in premenopausal women for LR2 of 85% and 91%, respectively, and for Simple Rules 93% and 83%, respectively.

Computer-Aided-Diagnosis (CAD) systems are gaining more interest in the last years, due to the great development of intelligent systems based on Machine Learning (ML), such as ANNs. These decision support tools can offer benefits over expert analysis, due to limitations of human examiners, and there are several research groups developing and improving them for multidisciplinary medical applications [12, 13]. With respect to characterization of ovarian tumors, a recent and valuable contribution based on ML systems has been published by Khazendar et al [14–16]. They have proposed a Support Vector Machine (SVM) model based on a decision level fusion, based on a database of 187 cases collected from the Department of Obstetrics and Gynecology of University Hospital KU, Leuven, Belgium. They analyzed gray-scale histograms, and local binary pattern histograms extracted from the features database, and constructed their decision level fusion strategy based on two main situations. The first situation is when both analysis characterize the tumor in the same diagnostic group (benign or malignant), then is accepted as correctly characterized, and the confidence level depends on each one of the analysis’s confidence. The second situation is when both features characterize the tumor in a different diagnostic group (one is benign and the other is malignant), and there is an uncertain decision, so that the image cannot be classified by this tool, except if one of the features is classified with high confidence level and the other is classified with low confidence level, so that tumor would be characterized in the high confidence level group but with a low global confidence level. They found 18.3% of images that could not be classified by this system, and the average accuracy using feature level fusion was 77%.

In order to extend the recent research works of Khazendar et al. [14–16], the objective of this paper is to provide a complementary study for the same ultrasound image database using Fourier Transform (FT) based feature descriptors as originally proposed in [16] and different well-known Machine Learning (ML) approaches for performing the classification stage of the ovarian tumors. Our aim is to find the best classifier using the FT, and as a novelty, the ELM algorithm has been used and compared with classical classifiers in this type of problem. This work is one of the first steps to be completed during the complex design of our CAD system for categorizing ovarian tumors using ultrasound images. Section 2 describes the material and methods, focusing in the two last stages of feature extraction and classification. Then, in Section 3, experimental results are shown and discussed. Finally, Section 4 ends this work with the main conclusions and future related research studies.

## Materials and methods

In this section, we describe the ultrasound image database, how these images were acquired and the feature extraction from them (Subsection 1), as well as the classification models we tested (Subsection 2), and how we performed evaluation and testing protocol (Subsection 3). S1 Fig shows the main stages of the implemented CAD system and it should be noted that this work is mainly focused on the last stage of classification.

In this research work, we use extracted features from real ultrasound images. The original ultrasound images collection is a selection from a 384 images pack collected by the University Hospital of the Catholic University of Leuven, in an original protocol approved by the Central Ethics Committee for Clinical Studies at the University Hospitals Leuven, Belgium, and by the local ethics committee at each recruitment centre as described in [10]. This original image collection was created by IOTA group to develop previous researches, and all participant gave written informed consent to use, analyse and publish the data. The selection of the images and the features extraction has been performed by the Buckingham University, granted ethical approval by the University of Buckingham’s School of Science Medicine Ethics Committee in May 2012, following the STARD guidelines for diagnostic accuracy studies. As our team works with extracted features provided by the Buckingham Universtity research group in a fully anonymized format, instead of human images, there is no need for approval from our local ethics committee. In particular, the analysed database includes extracted features from 187 ovarian tumour images taken in B-Mode, without Doppler signals, classified as benign (112 images) or malignant (75 images) depending on their pathological diagnosis post-surgery. The surgical operation was undertaken within a 120 days maximum period from the image acquisition, to get its pathological diagnostic as close as possible. As an example, S2 Fig shows two ultrasound images for benign and malignant ovarian tumors.

From these 187 images, two types of feature vectors were originally extracted [14–16]: Histograms of Intensity Features, and Local Binary Pattern Features. Both feature extraction methods have been done following four settings: Original image (extracting features without preprocessing the original image), Enhanced image (extracting features after preprocessing the original image by image enhancing techniques), Segmented Region of Interest (determining manually on each original image the interest areas, and then extracting features) and Segmented Region of Interest Enhanced (after enhancing the original image, determining manually the interest areas and then, extracting features). As we previously mentioned, this dataset has already been used by researchers from the Buckingham University [14–16] and they found that the best classification performance could be reached by means of features computed on the Segmented ROI Enhanced. Following this, in this research work, the Fourier Transform (FT) features are computed and, then, this information is used for training and evaluating ML classification models in order to measure its influence on the final performance of the implemented CAD system. The main aspects of the feature extraction stage based on FT are briefly described below in S3 Fig and **Algorithm 1**.

In image processing, FT is a mathematical tool used to decompose an image into its sine and cosine components [17]. The output we obtain is represented in the frequency domain (or Fourier domain), while the original input image is in the spatial domain. In the Fourier domain image, each point represents a frequency that is contained in the spatial original image. Fast Fourier Transform (FFT) is an efficient algorithm that allows calculating Discrete Fourier Transform (DFT) -as we are only concerned with digital images- and its inverse, obtaining a new image in the spatial domain. It should be noted that the FT computes a complex number values image, i.e. an image for the magnitude part and another image for phase part, and, in terms of image processing, only the magnitude of the FT is analyzed because it contains most of the information of the geometry of the spatial domain.

### How the FFT features are extracted: The Feature Extraction Algorithm

**Algorithm 1** The Feature Extraction Algorithm.

 1. Transform an image into frequency domain using FFT. Compute its power spectrum.

 2. Binarize the FFT power spectrum image using a trained threshold.

 3. Determine the best fit ellipse shape in the centre of the binary spectrum image.

 4. Extract the major diameter, minor diameter and the area of the shape in terms of the number of pixels, producing a 3D feature vector (major, minor, area).

#### Rationales behind the FFT Feature

The Discrete Fourier Transformation (DFT) is a signal analysis tool that decomposes time/space functions (such as images) into its different frequency waveform components in the same way prisms analyze sunlight into the rainbow of different colors. The DFT of an image f of size MN for any frequency pair (u, v) is a complex number that depends on all the pixel values f(x, y) computed by the formula:

$$\begin{array}{c} F\left(u,v\right)=[\frac{1}{MN}\sum_{x=0}^{M-1}\sum_{y=0}^{N-1}f\left(x,y\right)cos\left(2\pi \left(\frac{ux}{M}+\frac{vy}{N}\right)\right)-\frac{1}{MN}\sum_{x=0}^{M-1}\sum_{y=0}^{N-1}f\left(x,y\right)sin\left(2\pi \left(\frac{ux}{M}+\frac{vy}{N}\right)\right)] \end{array}$$ (1)

Since the transformation produces complex numbers, the output of the DFT transformation cannot be displayed as a single image. However, the polar representation of F(u, v) provides a more useful way of capturing information about the image features in terms of the spectrum of F defined as the modulus of F:

$$\begin{array}{c} ||F(u,v))||=\sqrt{{\left(Re\left(F\left(u,v\right)\right)\right)}^{2}+{\left(Im\left(F\left(u,v\right)\right)\right)}^{2}} \end{array}$$ (2)

and its phase:

$$\begin{array}{c} ⌀(F(u,v))=arctan(\frac{Im((u,v))}{Re((u,v))}) \end{array}$$ (3)

The Fourier spectrum by itself provides information about the strength of the image features especially in the directions of dominant discontinuities in the image (i.e. edges and other geometric texture features). These discontinuities are indicated by the highlighted rays radiating from the central frequency at (0, 0) which represents the total image energy. It is medically known that US scan images of malignant tumours tend to contain more details and complex structures compared to the much simpler images of benign tumours. Images listed in S4 Fig confirmed this observation when modelling the shape of low frequencies spectrum by the FFGF features. The S4 Fig shows that malignant images tend to have bigger/fatter ellipses i.e. more details and more complex structures compared with those of benign cases. The more textures the input image has, the more geometric features are involved, and consequently the more energy concentration in the central regions of the spectrum. The binarization of the FFT septum image using a sensible threshold produces an “elliptic” blob at the centre. The characterizing parameters of the best fit ellipse (i.e. major and minor axis, area, perimeter and orientation) capture the amount of spectrum energy concentration. In this paper we extract the triple (major axis, minor axis, area) to represent the input ultrasound images as one benign/malignant discriminating feature vector to be called the FFGF. As indicated by the example images in the table below, benign tumor images have less geometric discontinuities, and hence the elliptic shape obtained from the spectrum image tends to appear slimmer with high major/minor ratio. On the other hand, malignant tumor images have much more geometric discontinuities throughout the image, and the elliptic shape in the spectrum tend to be fatter with low major/minor ratio. In addition, the size of the elliptic shape is a good indicator of the amount of the geometric discontinuities. Therefore, these indicators collectively discriminate the benign tumors from the malignant ones.

### Classification stage

This section describes the main notions of the four ML classification methods analyzed in this work: *K-Nearest Neighbors* (KNN), *Linear Discriminant (LD)*, *Support Vector Machine* (SVM) and *Extreme Learning Machine (ELM)*. Note that, in this work, the obtained FFT Geometry features will be used as inputs during training and evaluating the four classifiers. Then, according to our notation, the dataset **X** is composed of *N* = 187 input vectors,

$$\begin{array}{c} X={\left\{x_{n}\right\}}_{n=1}^{N} \end{array}$$ (4)

where each input vector is composed of the three FFT Geometry features:

$$\begin{array}{c} x_{n}={\left[x_{n,1},x_{n,2},x_{n,3}\right]}^{T} \end{array}$$ (5)

In addition to this, the two possible classes of a given input vector (*C*_1_ and *C*_2_: benign and malignant) are respectively labeled with +1 and -1. Then, the target (or desired output) vector is denoted by

$$\begin{array}{c} t={\left\{t_{n}\right\}}_{n=1}^{N} \end{array}$$ (6)

where *t*_*n*_ could be +1 (*C*_1_: benign class) or -1 (*C*_2_: malignant class).

### K-Nearest Neighbors (KNN)

The KNN classifier assigns the input vector to that class having most training examples among the *K* neighbors of the input vector to be classified [18]. In the standard version of this non-parametric classifier, all neighbors have equal vote and the class having the maximum number of voters among the *K* neighbors is chosen. In this method, the value of *K* is the main parameter to be selected. Another important aspect is the suitable selection of the distance metric. This work considers two widely-used metrics: *Euclidean distance* and the *City block metric*. Both are special cases of the *Minkowski metric*. Given two different input vectors **x**_*n*_ and **x**_*m*_, their distance using the Minkowski metric is given by

$$\begin{array}{c} d_{n,m}=\sqrt[{p}]{\sum_{i=1}^{d}{\left|x_{n,i}-x_{m,i}\right|}^{p}} \end{array}$$ (7)

being *d* equal to three in this research work. The City block distance is a special case of the Minkowski metric, with *p* = 1; and when *p* = 2 we obtain the Euclidean distance. A disadvantage of the KNN method is that all the training data samples must be retained to classify future input instances.

### Linear Discriminant (LD)

The LD classifier obtains the class of **x** using a weighted linear combination of its input features [18, 19]:

$$\begin{array}{c} y\left(x\right)=w_{0}+w_{1}x_{1}+w_{2}x_{2}+...+w_{d}x_{d} \end{array}$$ (8)

where w = [*w*_1_, *w*_2_, …, *w_d_*] is the weight vector and *w*_0_ is the bias term. The magnitude of the weight *w*_*i*_ shows the importance of *x*_*i*_ and its sign indicates if the effect is positive or negative. In a LD, **x** is assigned to class *C*_1_ if *y*(**x**) > 0 and to class *C*_2_ if *y*(**x**) < 0. The decision boundary is given by those inputs that give *y*(**x**) = 0 and it is a (*d*-1)-dimensional hyperplane in the *d*-dimensional input space. Although its simplicity, LD has shown its usefulness in many real world applications [20]. In fact, it has been proved that the optimal discriminant is linear when the classes are Gaussians with a shared covariance matrix [18, 19]. The LD approach can be used even when this assumption does not hold and the weight parameters can be computed without making any assumptions on the class densities [18]. There are several techniques to determine suitable values for the weight parameters of a LD using the available training data [19]. In particular, this work applies the widely-used least squares approach that minimizes the following error function:

$$\begin{array}{c} E=\frac{1}{2}\sum_{n=1}^{N}{\left(y_{n}-t_{n}\right)}^{2}=\frac{1}{2}\sum_{n=1}^{N}{\left(\left(w_{0}+w_{n}^{T}x_{n}\right)-t_{n}\right)}^{2} \end{array}$$ (9)

and its ordinary least squares solution is given by

$$\begin{array}{c} \hat{w}={\left(X^{T}X\right)}^{-1}X^{T}t=X^{†}t \end{array}$$ (10)

where **X**^†^ is the Moore-Penrose generalized inverse matrix of **X**. Note that the Singular Value Decomposition (SVD) of **X** is used to compute the pseudoinverse for ensuring numerical stability and faster computations.

### Support Vector Machine (SVM)

Support Vector Machines (SVM) [18, 19] is a kernel-based discriminant method based on statistical learning theory. A kernel is a function that transforms the input data into a high-dimensional space and it can be linear (the dot product) and nonlinear (such as the gaussian or the polinomial) functions. According to our previous experience [14–16], a linear kernel has been chosen in this research study. After the input is transformed by applying the kernel, SVM determines the maximum margin hyperplane for separating the two different classes in the resulting high-dimensional space. Its analytical solution is given by convex optimization approaches. Several training methods have been proposed for SVM [19]. In this work, SVM classifiers are trained using two well-known procedures: Least Squares (LS) and Sequential Minimal Optimization (SMO).

### Extreme Learning Machine

The Extreme Learning Machine (ELM) is based on the concept that if the Multilayer Perceptron (MLP) input weights are fixed to random values, the MLP can be considered as a linear system and the output weights can be easily obtained using the pseudo-inverse of the hidden neurons outputs matrix **H** for a given training set. Although related ideas were previously analyzed in other works [21, 22], Huang was who formalized it, [23, 24]. He demonstrated that the ELM is an universal approximator for a wide range of random computational nodes, and all the hidden node parameters can randomly be generated according to any continuous probability distribution without any prior knowledge. Thus, given a set of *N* input vectors, an MLP can approximate *N* cases with zero error, $\sum_{i=1}^{N}\left||y_{i}-t_{i}|\right|=0$, being **y**_*i*_ ∈ *R*^*m*^ the output network for the input vector **x**_*i*_ ∈ *R*^*n*^ with target vector **t**_*i*_ ∈ *R*^*m*^. Thus, there exist *β*_*j*_ ∈ *R*^*m*^, **w**_*j*_ ∈ *R*^*n*^ and *b*_*j*_ ∈ *R* such that,

$$\begin{array}{c} y_{i}=\sum_{j=1}^{M}\beta_{j}f\left(w_{j}\cdot x_{i}+b_{j}\right)=t_{i},\;i=1,...,N. \end{array}$$ (11)

where *β*_*j*_ = [*β*_*j*1_, *β*_*j*2_, …, *β*_*jm*_]^*T*^ is the weight vector connecting the *j*th hidden node with the output nodes, **w**_*j*_ = [*w*_*j*1_, *w*_*j*2_, …, *w*_*jn*_]^*T*^ is the weight vector connecting the *j*th hidden node with the input nodes, and *b*_*j*_ is the bias of the *j*th hidden node.

For a network with *M* hidden nodes, the previous *N* equations can be expressed by

$$\begin{array}{c} HB=T, \end{array}$$ (12)

where

$$\begin{array}{l} \begin{array}{c} H\left(w_{1},\ldots ,w_{M},b_{1},\ldots ,b_{M},x_{1},\ldots ,x_{N}\right)= \end{array}\\ \begin{array}{c} ={\left[\begin{array}{ccc} f\left(w_{1}\cdot x_{1}+b_{1}\right) & \ldots & f\left(w_{M}\cdot x_{1}+b_{M}\right)\\ \vdots & \ldots & \vdots \\ f\left(w_{1}\cdot x_{N}+b_{1}\right) & \ldots & f\left(w_{M}\cdot x_{N}+b_{M}\right) \end{array}\right]}_{N\times M} \end{array} \end{array}$$ (13)

$$\begin{array}{c} B={\left[\begin{array}{c} \beta_{1}^{T}\\ \vdots \\ \beta_{M}^{T} \end{array}\right]}_{M\times m}\text{and}\;T={\left[\begin{array}{c} t_{1}^{T}\\ \vdots \\ t_{N}^{T} \end{array}\right]}_{N\times m} \end{array}$$ (14)

where **H** ∈ *R*^*N*×*M*^ is the hidden layer output matrix of the MLP, **B** ∈ *R*^*M*×*m*^ is the output weight matrix, and **T** ∈ ℜ^*N*×*m*^ is the target matrix of the *N* training cases. Thus, as **w**_*j*_ and *b*_*j*_ with *j* = 1, …, *N*, are randomly selected, the MLP training is given by the solution of the least square problem of (12), i.e., the optimal output weight layer is $\hat{B}=H^{†}T$, where **H**^†^ is the Moore-Penrose pseudo-inverse [25, 26].

Thus, ELM for training MLPs can be summarized as shown in **Algorithm 2**.

**Algorithm 2** Extreme Learning Machine (ELM).

**Require:** Given a training set $D=\{\left(x_{i},t_{i}\right)|x_{i}\in R^{n},t_{i}\in R^{m},i=1,\ldots ,N\}$, an activation function *f* and an hidden neuron number *M*.

 1. Assign arbitrary input weights **w**_*j*_ and biases *b*_*j*_, *j* = 1, …, *M*.

 2. Compute the hidden layer output matrix **H** using (13).

 3. Calculate the output weight matrix **B** = **H**^†^**T**, where **B** and **T** are both defined in (14).

ELM provides a fast and efficient MLP training [27], but it needs to fix the number of hidden neurons. In order to avoid the exhaustive search for the optimal value of *M*, several pruned methods have been proposed [28–33], among them, the most commonly used is is the ELM Optimally Pruned (OP-ELM) [31]. The OP-ELM sets a very high initial number of hidden neurons (*M* ≫ *N*) and, by using Least Angle Regression algorithm (LARS) [34], sorts the neurons according to their importance to solve the problem (12). The pruning of neurons is done using Leave-One-Out Cross-Validation (LOO-CV) by choosing that combination of neurons (which have been previously sorted by the LARS algorithm) that provides lower LOO error. The LOO-CV error is efficiently computed using the Allen’s formula [31].

### Performance evaluation and testing protocol

Four measures for performance evaluation [18] have been used: Accuracy (ACC), Area Under ROC Curve (AUC), Sensitivity (SEN) and Specificity (SPE). In one hand, accuracy is given by the following equation:

$$\begin{array}{c} ACC=\frac{TP+TN}{TP+FP+TN+FN} \end{array}$$ (15)

where TP, TN, FP and FN denote true positives, true negatives, false positives and false negatives, respectively. Traditionally, the most widely-used performance measure in classification problems is ACC. However, it ignores the probability estimations of classification in favor of class labels. In many research areas, and particularly biomedical applications, AUC provides an effective way to measure the overall performance of a classifier. AUC takes values from 0 to 1, where 0 indicates a perfectly inaccurate model and 1 reflects a perfectly accurate model. In general, a value of 0.5 for AUC is considered as the lower bound.

In order to make an accurate and fair performance evaluation of the different classification approaches, this work uses a Leave-One-Out Cross Validation (LOO-CV) procedure [18]. LOO-CV avoids undesirable shifts from the random selection of training and test sets. For the *N* total number of samples involved in the study, one is retained for testing, and the remaining *N*-1 are used for training the classifier. This process is repeated *N* times (i.e. an iteration for each input vector). Note that all cases are used for training and testing purposes during the *N* iterations of the LOO-CV procedure and, also, the performance evaluation measures are computed at the end of this iterative procedure. For the ELM, the LOO-CV procedure is performed 30 times, due to the random initialization of its weights, this results are shown in terms of best result (Table 1) and mean and standard deviation (Table 2).

**Table 1:** Obtained results (Accuracy (in %) -ACC-, Area under ROC curve -AUC-, sensitivity (in %) -SEN- and specificity (in %) -SPE- in%) by KNN, LD, SVM and ELM classifiers using FFT Geometry features. Performance evaluation has been done under a LOO-CV procedure.

Method		ACC	AUC	SEN	SPE
KNN	Euclidean distance, K = 1	50.27	0.4836	58	40
	Euclidean distance, K = 10	52.94	0.4522	78	16
	Euclidean distance, K = 15	56.68	0.4377	91	5
	Euclidean distance, K = 30	55.08	0.3907	89	4
	City block distance, K = 1	53.48	0.5127	63	40
	City block distance, K = 10	57.20	0.5169	82	20
	City block distance, K = 15	58.29	0.4912	93	7
	City block distance, K = 30	58.29	0.4801	94	5
LD	Least Squares method	85.56	0.8514	89	80
SVM (with Linear kernel)	SMO training	87.70	0.8740	91	83
	LS training	86.10	0.8545	88	84
ELM (best result)	Linear Kernel	84.49	0.8551	94	71
	Sigmoid Kernel	87.17	0.8676	90	80
	Gaussian Kernel	86.10	0.8620	92	79
	Linear-Sigmoid kernel	86.10	0.8553	90	80
	Linear-Gaussian kernel	87.70	0.8740	92	80
	Sigmoid-Gaussian kernel	87.17	0.8692	93	79
	Linear-Sigmoid-Gaussian kernel	87.17	0.8765	93	77

**Table 2:** Obtained results (Accuracy -ACC-, Area under ROC curve -AUC- and Hidden neurons -HN-) by ELM classifier of several kernels (in terms of mean and standard deviation) using FFT Geometry features. Performance evaluation has been done under a LOO-CV procedure.

Kernel	ACC (in %)	AUC	HN
Linear	84.49±0.00	0.8551±0.0000	3.00±0.00
Sigmoid	82.16±1.87	0.8183±0.0200	15.21±0.48
Gaussian	84.90±0.98	0.8486±0.0109	15.70±0.41
Linear-Sigmoid	82.37±1.62	0.8199±0.0174	15.82±0.48
Linear-Gaussian	85.22±1.17	0.8513±0.0125	16.11±0.44
Sigmoid-Gaussian	82.82±2.05	0.8260±0.0217	13.75±0.45
Linear-Sigmoid-Gaussian	82.30±2.04	0.8208±0.0214	13.40±0.37

## Results and discussion

Experiments have been carried out in MATLAB R2018a environment running in the same machine. Table 1 shows the obtained results, in terms of four measures of classification performance: Accuracy, Area Under ROC Curve, Sensitivity and Specificity. As a first comment, it is possible to see from this table that the KNN classifier provides very poor performance in this problem, independently of the chosen distance (Euclidean or City block) and the size of the local approximation (i.e. the number of nearest neighbors). Although larger values of K and the City block distance give an increased performance, it is still very poor (less than 59% of accuracy). This can be explained because FFGF acts as an effective dimension reduction method, with a little loss of information, so, the Euclidean distance of the Nearest Neighbour is not an appropriate method to classify it, due to the relatively high dimension of the vectors. Therefore, and according to these obtained results, KNN should be omitted for the design of the classification stage of our CAD system. With respect to the three other classifiers, LD, SVM and ELM, its performance results are significantly higher than those obtained with KNN. Comparing them, ELM and SVM give better ACC and AUC than LD and these advantages are clearer when the kernel-based discriminant given by the SVM method is trained using the Sequential Minimal Optimization approach and Sigmoid-Gaussian kernel for the ELM.

Finally, and considering that the previously obtained classification results with other feature descriptors (Histograms of Intensity Features and Local Binary Pattern Features) from the same image database was around 77.0% accuracy [14–16], the analyzed methods of this paper are able to provide an important enhancement in the performance: it achieved up to 87.7% of accuracy. In particular, and due to the fact SVM has been also applied in the previous studies [14–16] under the same LOO-CV procedure, the better performance is because the resulting feature information from the FFT Geometry descriptors makes the classification task easier. ELM (with Linear-Gaussian Kernel) provides a similar result (ACC and AUC) to SVM.

Some developed tools with a high performance results, as IOTA’s Linear Regression model 2 (LR2), include not only the image analysis but clinical data too [11]. When using LR2, the clinician has to analyze if the image has presence of ascites, papillary projections, acoustic shadows, irregular internal cyst walls, Doppler signaling captation within a solid papillary projection, and take in account the patient’s age and maximal diameter of the solid component. The system gives a different weight to these parameters, and the result is a probability of malignancy to the studied image, but it doesn’t classify if the image is benign or malignant (just gives a probability of being benign or malignant).

Database used in this work is composed by different nature ovarian tumours images, from benign to malignant (stromal tumours, epithelial tumours, metastatic tumours or embrionary tumours). Classifying these images is a daily challenge for the clinician, who often can feel doubts with not clearly benign or malignant images, finding a high interpersonal variation when examining the same case. Our method includes all kind of ovarian tumour images, even some difficult to classify images, that are normally the clinically more interesting to characterize.

Our results are similar to human performance [4, 5], with a high Sensitivity (92%) and Specificity (80%) when using ELM, taking in account that we don’t use any clinical data from the patient, and we only classify B-mode ultrasound images, without any Doppler signaling, that could improve the results, as other methods do to modulate the image weight in the classification process.

Used algorithms in this work have a low computing weight, so, it could be an advantage to their implementation on medical devices, so it could be helpful for the clinician in high difficulty classification cases, as well as it could be used during fellowship training.

By the moment, there is no developed CAD system based in Artificial Intelligence incorporated to medical Ultrasound scanner systems, or widely used for fellow’s training. Our team want to remark that Artificial Intelligence has its place in this field, and can be used as a tool to help clinicians to classify difficult tumour images, or help them in their medical training.

## Conclusions and future Work

ML methods can be efficiently used for developing the classification stage in computer-aided diagnosis systems based on ultrasound images of ovarian tumors. In particular, LD and SVM approaches are able to provide automatic classification with a high accuracy. Besides, and according to our obtained results, FFT Geometry descriptors from the ultrasound images provide relevant and useful information to classify ovarian tumors. Future work should aim at enhancing the classifier design using other learning approaches, such as the Extreme Learning Machine (ELM) algorithm and its variants, and the application of ensemble techniques. Another ongoing work is to exploit and combine different kind of features extracted from ultrasound images.

## Supporting information

S1 Fig Stages of the CAD system. (1) Acquisition of ultrasound images; (2) Dataset construction; (3) Classification.
(EPS)

S2 Fig Examples of ultrasound images from the database. (a) Benign ovarian tumor; (b) Malignant ovarian tumor.
(EPS)

S3 Fig Steps involved (from left to right). Ultrasound image of Ovarian tumor, Fourier spectrum of the tumor, threshold of the spectrum and best fitted ellipse.
(EPS)

S4 Fig Comparing spectrum of ultrasound scans of benign and malignant ovarian tumors. (EPS)
